# Pathogenesis of bronchopulmonary dysplasia: when inflammation meets organ development

**DOI:** 10.1186/s40348-016-0051-9

**Published:** 2016-06-29

**Authors:** Tayyab Shahzad, Sarah Radajewski, Cho-Ming Chao, Saverio Bellusci, Harald Ehrhardt

**Affiliations:** 1Department of General Pediatrics and Neonatology, Center for Pediatrics and Youth Medicine, Justus-Liebig-University, Feulgenstrasse 12, D-35392 Gießen, Universities of Gießen and Marburg Lung Center (UGMLC), Member of the German Lung Research Center (DZL), Giessen, Germany; University of Giessen Lung Center, Excellence Cluster Cardio-Pulmonary Systems, Member of the German Lung Center, Department of Internal Medicine II, Aulweg 130, 35392 Giessen, Germany

**Keywords:** Bronchopulmonary dysplasia, Chronic lung disease of the preterm infant, Lung development, Inflammation, Apoptosis, Alveologenesis, Vasculogenesis, Infection, Antenatal steroids, Surfactant

## Abstract

Bronchopulmonary dysplasia is a chronic lung disease of preterm infants. It is caused by the disturbance of physiologic lung development mainly in the saccular stage with lifelong restrictions of pulmonary function and an increased risk of abnormal somatic and psychomotor development. The contributors to this disease’s entity are multifactorial with pre- and postnatal origin. Central to the pathogenesis of bronchopulmonary is the induction of a massive pulmonary inflammatory response due to mechanical ventilation and oxygen toxicity. The extent of the pro-inflammatory reaction and the disturbance of further alveolar growth and vasculogenesis vary largely and can be modified by prenatal infections, antenatal steroids, and surfactant application.

This minireview summarizes the important recent research findings on the pulmonary inflammatory reaction obtained in patient cohorts and in experimental models. Unfortunately, recent changes in clinical practice based on these findings had only limited impact on the incidence of bronchopulmonary dysplasia.

## Introduction

**Fig. 1:**
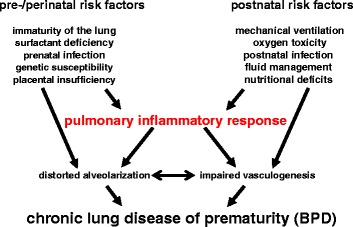
Central risk factors for BPD development. Depicted are the central pre-/perinatal and postnatal risk factors contributing to the pathogenesis of BPD

Bronchopulmonary dysplasia (BPD) is a chronic lung disease of preterm infants. The current worldwide used classification takes into account the need for mechanical ventilation and oxygen supplementation at 28 days of life and at 36 weeks of gestation. Despite major treatment advances during the last two decades, the incidence of BPD is still above 30 % in preterms below 30 weeks of gestation in most European countries [1]. Extremely preterm infants are delivered in the saccular stage of lung development. BPD is caused by the disturbance of lung development in this critical period. The diagnosis is associated with lifelong restrictions of pulmonary function and increases the risk for abnormal somatic and psychomotor development [2]. The inflammatory alterations observed in preterms developing BPD are restricted to the neonatal period, but the pulmonary metabolomic abnormalities persist into adulthood. Current animal experiences raise fears that former preterms will develop a COPD-like phenotype later in life with all the consequences for quality of life and life expectancy [3–6]. The factors contributing to this disease’s entity can be separated into pre-, peri-, and postnatal causes. Within the ante- and perinatal factors, genetic susceptibility, the immaturity of the surfactant homeostasis, intrauterine and perinatal infections, and lung growth restriction due to placenta insufficiency are central factors impacting on the development of BPD. The postnatal lifesaving therapies of mechanical ventilation and oxygen therapy induce a pulmonary inflammatory response. Lung development is further affected by fluid overload and nutritional deficits (Fig. 1). Despite the progress in the mechanistic understanding of the pathogenesis of BPD, the therapeutic options to prevent this disease are still limited and drug therapies are of low efficiency resulting in an only modest reduction of BPD incidence [7, 8].

We will discuss in detail the recent advances in the understanding of physiologic lung development and the central contribution of the pulmonary inflammatory response to the disturbance of this highly orchestrated process. The potentials and limitations of established and new therapeutic strategies are discussed based on recent preterm cohort studies.

## Alterations of normal lung development

### Stages of lung development

Physiologic lung development is a highly orchestrated process which in the end enables the gas exchange between the air-conducting parts of the lung and the blood vessels. The stages of lung development can be divided into the embryonic, the pseudoglandular, the canalicular, the saccular, and the alveolar stage. During the embryonic stage, the lung bud separates from the gut followed by the branching morphogenesis in the pseudoglandular stage until 17 weeks of gestation. The pseudoglandular stage is followed by the canalicular stage which continuous until the border of viability at about 24 weeks. The canalicular stage is characterized by the formation of the terminal branches of the bronchial tree, differentiation of type I and type II cells, vascular outgrowth, and the thinning of the mesenchyme. The subsequent saccular stage is mainly characterized by the formation of the primitive terminal airspaces, thinning of the connective tissue between the airspace and the pulmonary vessels, and the beginning of surfactant production. The saccular stage is marked by a dramatic improvement of the prerequisites for gas exchange, and any derangement of this vulnerable period will lead to relevant limitations of gas exchange.

### Consequences of disruption of lung development in the saccular stage

Premature birth in the early phase of the saccular stage leads to disruption of lung development and impaired septation. The pro-inflammatory pulmonary response which is provoked by infection, mechanical ventilation, and oxygen therapy negatively impacts these critical steps which results in a reduced number of alveoli, reduced surface area for gas exchange, and simplified alveolar structures. These impairments have dramatic consequences for gas exchange, and many preterms need prolonged periods of mechanical ventilation, respiratory support, and oxygen delivery. The further lung development during the alveolar stage cannot induce a catch-up growth which leads to relevant limitations of lung function persisting into adulthood [9, 10]. The impairments of development of the air-conducting parts of the lung are accompanied by a more or less serious distortion of pulmonary vascular development. This double pathology was confirmed in lung tissue sections from patients with severe BPD and leads to the clinical situation of a double limitation of gas exchange due to a restriction of the lung surface area and a reduced capillary network. More severe restrictions of lung function run a higher risk to develop pulmonary hypertension which is of clinical relevance in up to 25 % of preterms with the diagnosis of BPD [11].

### Central signaling pathways for alveolar and vascular development

For the molecular understanding of alveolar and vascular lung development, we have to rely on the data from animal models which clearly demonstrate that alveolar development cannot be uncoupled from vascular development. The proper composition of the extracellular matrix in between is essential for the critical steps of lung developmental, and both alveolar and vascular growth requires the interaction with the extracellular matrix. Vascular endothelial growth factor A (VEGFA) is a key player of vascular development and mediator of NO synthesis in the endothelium. Its inhibition is accompanied by an inhibition of alveologenesis in several animal species [12]. The HIF signaling pathway is a further central regulatory pathway of vascular development, and its dysregulation leads to the distortion of vascular development dependent and independent of VEGFA [13, 14]. Cytokines of the inflammatory response like MCP-1 or MIP-1α are able to derange the formation of alveolar capillaries [15]. The parallel development of alveolar and microvascular structures is highly orchestrated by the extracellular matrix which forms the interlayer in between. The development of the secondary septae depends on the proper assembly of elastin fibers at specific sites which is deranged in the pathologic situation. In line with this, elastin haploinsufficiency leads to the distortion of the mesenchyme and of vascular development [16]. In the pathologic situation of lung injury, these fibers are excessively and diffusely deposited in the mesenchyme. Mechanical ventilation leads to inappropriate deposition and elastin breakdown accompanied by distortion of further septation [17–19]. Proper elastin fiber deposition is orchestrated by the fibroblasts in the interstitial mesenchyme, and different genetic knockout models have proven that the loss of fibroblasts is associated with severe derangement of normal lung development and remodeling of the extracellular matrix. In animal models mimicking the clinical situation of mechanical ventilation and oxygen therapy in the saccular stage of lung development, the fibroblasts and elastic fibers are diffusely scattered in the mesenchyme and are no longer located at the top of the secondary crests where they normally contribute to further lung growth [20, 21]. The inhibition of the C-terminal Src kinase (Csk) and the subsequent overweight of epidermal growth factor receptor signaling seem to be critical for the abnormal distribution of lung fibroblasts [22]. Within the further pathways which are critical for normal lung development, two vitamin-dependent signaling pathways have raised special attention: While the retinoic acid pathway is a well-known key regulator of critical steps of lung development, latest data hint that vitamin D is another positive regulator in the pathologic situation of lung damage [23–25].

## The pulmonary inflammatory response

### Imbalance of pro-inflammatory cytokines and growth factors

Since the early 1990s, it is well known that the pulmonary inflammatory response due to mechanical ventilation and oxygen therapy is characterized by a disbalance of pro-inflammatory cytokines and growth factors followed by the influx of inflammatory cells into the lung. Since the first observations of cytologic changes in the tracheal aspirates of preterms developing BPD, a plenty of studies has detected an association between higher levels of typical pro-inflammatory cytokines like IL-1β, IL-6, IL-8, TNF-α, monocyte chemo-attractant proteins, and macrophage inflammatory proteins in the tracheal aspirate of the mechanically ventilated preterm infant and the later development of BPD [26, 27]. The initiation of the inflammatory response can already occur in utero, i.e., in the situation of chorioamnionitis [28]. Further evaluation of these markers of inflammation in the tracheal aspirate and peripheral blood of the preterm infant is necessary to confirm the applicability as early biomarkers of disease severity [29, 30]. The rise in pro-inflammatory cytokines is accompanied by the upregulation of cell adhesion molecules like ICAM-1 and L-selectin and by the increase in chemotactic proteins that attract the inflammatory cells into the lung. The pulmonary attraction of these cells leads to the persistence of the inflammatory response and the accumulation of NF-kB within this cellular fraction. In parallel, the levels of classical anti-inflammatory cytokines like IL-10 and of central growth factors of alveolar and vascular growth like VEGFA and PDGFA and the crucial members of the FGF family are decreased in tracheal aspirates and preterm lung tissue sections [31–33]. A certain specificity of the inflammatory reaction can be attributed to the fact that the cytokine levels of not all classical candidates are significantly regulated including IL-4 and IL-13. Of special interest is the focus on cytokines and proteins which regulate normal lung development and participate in tissue remodeling. TGF-β and the BMP signaling pathways take a central position with respect to both processes. The activity of TGF-β is significantly increased in the tracheal aspirates of preterm infants which later develop severe BPD, and the appearance of α-SMA and TGF-β positive fibroblasts is increased in the alveolar septae of preterms with BPD [34, 35]. The expression level of matricellular protein SPARC is significantly elevated in tracheal aspirates and lung tissue sections from preterms with severe BPD which regulates cell-matrix interaction and participates in tissue remodeling among other regulatory steps by impacting on TGF-β, β-catenin and VEGFA signaling [36, 37]. Downstream of TGF-β, tissue transglutaminase-2 and lysyl hydroxylase plod2 were identified as critical regulators of extracellular matrix remodeling [38, 39].

### Influx of inflammatory cells into the lung

The cellular fraction in the tracheal aspirates is dominated by alveolar macrophages which contribute to the persistence of the inflammation by the production of further pro-inflammatory cytokines and by neutrophils which secrete a plenty of proteases, leading to pulmonary tissue damage, cellular apoptosis, and surfactant inactivation. Recent observations suggest that the immaturity of the macrophage phenotype may account not only for the severity of postnatal respiratory distress but also for the progression to BPD [40]. There seems to be a preexisting disbalance between proteinases and proteinase inhibitors in the developing lung which makes it more susceptible to organ damage. The ex utero higher oxygen pressures and the clinical need for increases in oxygen fraction induce further cell damage by the production of reactive oxygen species which cause lung damage by direct lipid peroxidation and aggravate the tissue damage by proteases. The increase of matrix metalloproteinases, cysteine proteases, elastase, and trypsin in the tracheal aspirates of preterms developing BPD suggests an important role in the pathogenesis. Data from clinical studies prove the association between the extent of the pulmonary inflammation and the increase in elastase activity in the tracheal aspirates of preterm infants later developing severe BPD. The elastin breakdown products like desmosine can be detected in higher levels in the urine of preterms developing severe BPD and may serve as early markers of disease progression [41, 42].

**Fig. 2:**
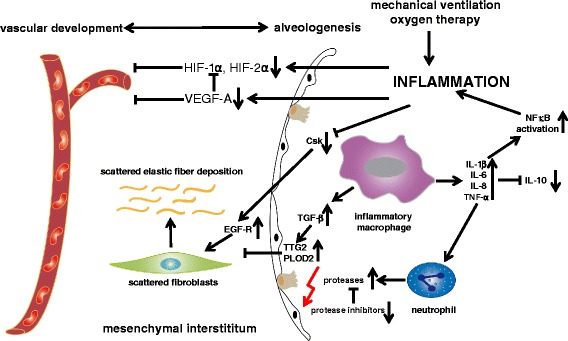
Pathogenesis of BPD: impact of the pulmonary inflammatory response on the premature lung. The scheme summarizes the effects of the pulmonary inflammatory response on alveolar and vascular development and the central components of lung development in the mesenchymal interstitium

Recent publications suggest that further cellular fractions like mast cells accumulate in the lung tissue and that reactive T cells in the peripheral blood also impact on the development of BPD. Their precise role remains to be determined. The phenomenon of immune tolerance or immunoparalysis due to antenatal infection has to be taken into account when evaluating the impact of hematopoietic inflammatory cells on distortion of lung development [43, 44]. The complexity of the interaction of the immune system with the microbial pathogens probably accounts for the contradictory results of the effects of pathogen colonization and infection on the development of BPD [44–46]. Experimental evidence underlines the importance of the order of exposures which determines the impact on lung inflammation [47]. The lifesaving therapies of mechanical ventilation and oxygen application have a direct impact on the different cell fractions of the lung. For example, the structure and function of airway smooth muscle cells is impacted by hyperoxia in a dose-dependent manner [48]. Besides the pro-inflammatory hematopoietic cells, mesenchymal stromal cells can be detected within the tracheal aspirates. The current scientific findings suggest a negative impact of these cells on the incidence of BPD as they are mainly isolated from tracheal aspirates of preterms with severe BPD and show higher β-catenin activation [37, 49]. Caution has to be taken before final conclusions are drawn as in other lung disease entities their precise function is still of controversial debate. The critical steps of inflammation are summarized in Fig. 2.

### The complexity of signal transduction

Animal models were able to reproduce the negative impact of pro-inflammatory cytokines like IL-1β on lung development in the saccular stage and to attribute the activation of the NF-kB signaling a central role in the pathogenesis of BPD. This is further fundamented by experimental data that demonstrate a beneficial effect of attenuation of NF-kB activation on lung development. However, the therapeutic potential of NF-kB inhibition is limited by its essential role in physiologic lung development, mediating both alveologenesis and vasculogenesis. Furthermore, a disease-limiting function of NF-kB in inflammation was attributed to the suppression of macrophage inflammatory protein 2 [50–53]. The physiologic functions of NF-kB in the developing lung are even more complex as it is ubiquitously expressed; controls diverse cellular functions including apoptosis, survival, proliferation, and immune regulation; and exerts simultaneously pro- and anti-inflammatory actions. In line with this, recent data ascribe TNF-α, a classical activator of NF-kB signaling, an important role for the limitation of overwhelming TGF-β signaling, for the protection from ventilator-induced lung injury and the preservation of lung development [54]. These caveats are further strengthened by the inconsistent results of glucocorticoid actions on the developing lung which are applied to suppress the inflammatory response. Despite their potent anti-inflammatory activity, contradictory effects of glucocorticoids were observed during saccular lung development. On the one hand, they promoted the expansion of distal airways and thinning of the mesenchyme but on the other hand impaired secondary septation [55]. Also for other key regulators like MMP-9, the data sets are not consistent and the impact has to be studied within the complexity of the disease [56, 57]. The appropriate level of TGF-β signaling is another example, and the reduction or the abundance of activation of TGF-β pathways is associated with distortion of lung development in any stage [58, 59]. Further complexity arises from differences in clinical practice and, i.e., the course of oxygen support. Recent experimental data hint that not only the duration and fraction of oxygen supply but also the change in oxygen fraction account for differences in disturbance of lung development [60, 61].

## Improvements in clinical care

### Moderate reduction of BPD incidence by modification of ventilation strategies

During the recent years, several clinical approaches have been tested to reduce the incidence of BPD. Several randomized controlled multicenter studies have tested the impact of different ventilation strategies to reduce the incidence of BPD: The comparison of initial stabilization of the preterm infant after delivery with CPAP versus intubation and surfactant application did not show inferiority of the CPAP without surfactant application strategy, but it did not significantly impact on the incidence of BPD [62, 63]. However, the follow-up at the age of 2 years did demonstrate superiority of the CPAP approach with respect to the number of infants with wheeze episodes, respiratory illnesses, and emergency visits [64]. In line with this, the comparison of high-frequency oscillation (HFO) versus conventional ventilation did show superiority of HFO and better lung function at the age of 11–14 years although again the BPD incidence at 36 weeks was not significantly different [65]. These studies underline that long-term follow-up is essential to precisely assess the impact on lung development. Whether the use of a more gentle ventilation strategy tolerating higher pC0_2_ levels impacts on the long-term pulmonary outcome is still not clear. The short-term pulmonary outcome at 36 weeks of gestation did not lead to a significant reduction in BPD [66]. Besides modification of the ventilation regime, the allocation to different oxygen saturation targets was extensively studied during the recent years. The meta-analysis of the recent multicenter trials with overall more than 5000 infants did not detect a significant difference for the outcome parameter BPD between oxygen saturation limits of 85–89 % versus 90–95 %. Here again, long-term follow-up is urgently needed. The detailed view on all relevant outcome parameters of prematurity revealed that the higher survival rates in the higher Sp0_2_ target group were accompanied by a lower incidence of necrotizing enterocolitis [67]. Therefore, it is essential to take into account all severe complications of preterm birth when comparing different ventilation strategies.

### Lack of new drug therapies to prevent BPD

More than 40 different therapeutic approaches have been tested in randomized controlled trials during the last two decades, but only 4 medications have proven therapeutic efficiency in meta-analyses. Also natural surfactant constitutes a backbone of therapy of respiratory distress of the preterm infant; its impact on the pulmonary outcome gets only visible in the meta-analysis when the parameter BPD is combined with death before discharge [68]. The two initial randomized controlled trials comparing the avoidance of mechanical ventilation by less invasive surfactant application under spontaneous breathing with clinical routine surfactant application via the endotracheal tube were probably underpowered to detect a significant difference with respect to the outcome parameter BPD [69, 70]. The actual retrospective analysis of a bigger cohort revealed that the sophisticated technique of less invasive surfactant application under spontaneous breathing led to a significant reduction in BPD incidence and overall morbidity [71]. Besides surfactant therapy, both the therapy with caffeine and the intramuscular application of vitamin A significantly reduce the incidence of BPD; also, the mechanisms leading to this reduction are not completely determined in the preterm infant. Despite the therapeutic efficiency to reduce the incidence of BPD the routine use of corticosteroids should be avoided because of the potential side effects on the psychomotor outcome and the potential risks for lung development [55]. Taking into account that efficient new therapeutic drugs to reduce the incidence of BPD are not within reach, the critical evaluation of these well-established drugs might lead to a reduction of BPD in the short term. On the other hand, a plenty of therapeutic strategies that have proven efficient in animal studies displayed no superiority in randomized controlled trials in the preterm infant. One of the major disappointments is the application of inhaled nitric oxide (iNO) during the initial phase of mechanical ventilation. Although iNO proved highly efficient in several animal studies and reduced the pulmonary inflammation, stabilized the surfactant homeostasis, and promoted lung growth, the results of the combined analysis of published randomized trials do not allow a recommendation of the use of iNO in the clinical setting [72].

## Conclusion

The latest data confirm that BPD is not only a multifactorial but a highly complex disease. The precise evaluation of signal transduction and pathway interactions will contribute to a thorough understanding of disease pathology. Investigations of both pathway activation and blockade of signal transduction are necessary for the determination of pathway-specific effects, and the readouts should focus on all pathways relevant for normal lung development and lung injury to get a comprehensive view on BPD. This research direction will hopefully overcome the current gap between progress in the molecular understanding of the pathologic alterations in animal models and the only limited advances to reduce the incidence of chronic lung disease in the preterm infant. Future studies are needed which comprehensively study the pathologic processes induced by premature birth, infections, mechanical ventilation, and oxygen therapy. As patient lung tissue samples are not readily available due to the high survival rates of preterm infants and the special disease severity of preterms not surviving till discharge, the search for readily available biomarkers to predict disease severity and to control therapy efficiency in animal and human trials is urgently needed. Furthermore, more specific and precise approaches are necessary to discriminate the different pathologies of BPD which take into account the different causes like antenatal growth restriction, genetic disposition, gender-specific effects, pre- and postnatal infection, and the therapeutic necessities of mechanical ventilation and oxygen toxicity. When considering these variables, a better patient-oriented therapeutic approach is possible which can improve therapeutic efficiency based on the molecular understanding of the different disease pathologies of bronchopulmonary dysplasia. The current need for long-term follow-up can be hopefully avoided by the introduction of more precise parameters to estimate disease severity beyond the dependence on mechanical ventilation and oxygen supplementation at 36 weeks of gestation.

## Abbreviations

BPD, bronchopulmonary dysplasia; COPD, chronic obstructive pulmonary disease; CPAP, continuous positive airway pressure; Csk, C-terminal Src kinase; FGF, fibroblast growth factor; HFO, high-frequency oscillation; ICAM-1, intercellular adhesion molecule-1; IL, interleukin; iNO, inhaled nitric oxide; MCP-1, monocyte chemotactic protein 1; MIP-1α, macrophage inflammatory proteins; MMP-9, matrix metallopeptidase 9; NF-kB, nucleic factor-kB; NO, nitric oxide; pCO2, partial pressure of carbon dioxide; PDGFA, platelet-derived growth factor subunit A; SPARC, secreted protein acidic and rich in cysteine; SpO2, peripheral capillary oxygen saturation; TGF-β, transforming growth factor β; VEGFA, vascular endothelial growth factor A; α-SMA, α-smooth muscle actin
